# Explainable convolutional neural networks for assessing head and neck cancer histopathology

**DOI:** 10.1186/s13000-023-01407-8

**Published:** 2023-11-03

**Authors:** Marion Dörrich, Markus Hecht, Rainer Fietkau, Arndt Hartmann, Heinrich Iro, Antoniu-Oreste Gostian, Markus Eckstein, Andreas M. Kist

**Affiliations:** 1 Department Artificial Intelligence in Biomedical Engineering, Friedrich-Alexander-Universität Erlangen-Nürnberg (FAU), Erlangen, 91052 Germany; 2 Department of Radiotherapy and Radiation Oncology, Saarland University Medical Center, Homburg/Saar, 66421 Germany; 3 Department of Radiation Oncology, University Hospital Erlangen, Friedrich-Alexander-Universität Erlangen-Nürnberg (FAU), Erlangen, 91054 Germany; 4 Comprehensive Cancer Center EMN, University Hospital Erlangen, Friedrich-Alexander-Universität Erlangen-Nürnberg (FAU), Erlangen, 91054 Germany; 5 Institute of Pathology, University Hospital Erlangen, Friedrich-Alexander-Universität Erlangen-Nürnberg (FAU), Erlangen, 91054 Germany; 6 Bavarian Cancer Research Center (BZKF), Bavaria, Germany; 7 Department of Otolaryngology - Head and Neck Surgery, University Hospital Erlangen, Friedrich-Alexander-Universität Erlangen-Nürnberg (FAU), Erlangen, 91054 Germany

**Keywords:** Explainable AI, Histopathology, Head and neck cancer, Semantic segmentation, Classification

## Abstract

**Purpose:**

Although neural networks have shown remarkable performance in medical image analysis, their translation into clinical practice remains difficult due to their lack of interpretability. An emerging field that addresses this problem is Explainable AI.

**Methods:**

Here, we aimed to investigate the ability of Convolutional Neural Networks (CNNs) to classify head and neck cancer histopathology. To this end, we manually annotated 101 histopathological slides of locally advanced head and neck squamous cell carcinoma. We trained a CNN to classify tumor and non-tumor tissue, and another CNN to semantically segment four classes - tumor, non-tumor, non-specified tissue, and background. We applied Explainable AI techniques, namely Grad-CAM and HR-CAM, to both networks and explored important features that contributed to their decisions.

**Results:**

The classification network achieved an accuracy of 89.9% on previously unseen data. Our segmentation network achieved a class-averaged Intersection over Union score of 0.690, and 0.782 for tumor tissue in particular. Explainable AI methods demonstrated that both networks rely on features agreeing with the pathologist’s expert opinion.

**Conclusion:**

Our work suggests that CNNs can predict head and neck cancer with high accuracy. Especially if accompanied by visual explanations, CNNs seem promising for assisting pathologists in the assessment of cancer sections.

**Supplementary Information:**

The online version contains supplementary material available at 10.1186/s13000-023-01407-8.

## Introduction

Head and Neck Squamous Cell Carcinoma (HNSCC) is a malignancy that can develop in several regions, such as the oral cavity, pharynx, or larynx [1]. Worldwide, HNSCC was the seventh most common cancer in 2020 [2]. HNSCC patients have a poor prognosis, and their disease is often diagnosed in an advanced stage [1]. Although the 5-year survival has improved over the last decades [1], it is still very low, ranging between 25% and 60% [3].

HNSCC is diagnosed by pathologists who assess tissue sections and provide important information for treatment choice and prognosis. Thin tissue slices are stained and evaluated using light microscopes, but recently can also be digitally analyzed as Whole Slide Images (WSIs). As WSIs are high-quality imaging data, many computer vision algorithms are being developed to reduce the workload of pathologists and improve the accuracy of the diagnosis.

Artificial Intelligence (AI) has seen a lot of attention recently, also in the medical field. Especially Convolutional Neural Networks (CNNs) have shown remarkable performance in the analysis of medical images, including WSIs [4]. However, translating AI systems into clinical practice remains difficult due to their black-box nature. Algorithms applied in the diagnosis of cancer need to be very reliable and trustworthy. Explainable AI methods can be used to tackle this problem and improve the transparency of neural networks, for example by offering visual explanations of predictions [5]. Explainable AI techniques help both developers and physicians to better understand AI algorithms, their abilities, and their limitations [6].

We aimed to investigate the ability of CNNs to classify and semantically segment head and neck cancer tissue. To this end, we manually annotated tissue in the WSIs in two distinct classes, namely tumor and non-tumor, and applied state-of-the-art CNNs. Additionally, we aimed to explore which features were responsible for both networks’ decisions, using the two Explainable AI methods Grad-CAM [7] and HR-CAM [8].

## Methods

### Data source

**Fig. 1:**
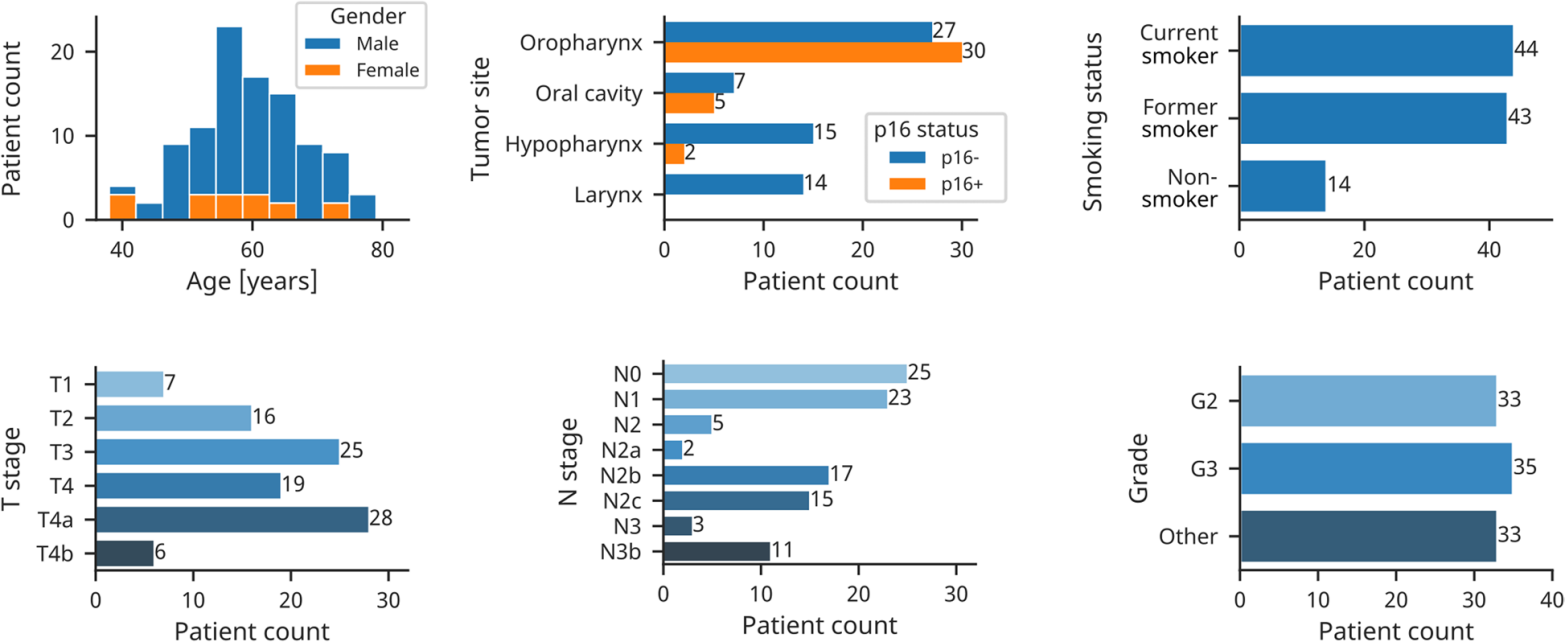
Characteristics of 101 head and neck cancer patients from the CheckRad-CD8 trial. Tumor stages are given according to the UICC TNM eighth edition. Grade “Other” denotes patients with HPV-positive oropharyngeal tumor or missing tumor grade. TNM = tumor-node-metastasis

The histopathological slices were collected in the context of the CheckRad-CD8 trial [9, 10]. In this trial, a cancer treatment that consists of induction therapy followed by radioimmunotherapy was developed [10]. Patients with locally advanced HNSCC of the oral cavity, oropharynx, hypopharynx, or larynx were selected in eight clinical centers in Germany [10]. Their diagnosis was confirmed by a biopsy of the primary tumor. The patients first received induction chemoimmunotherapy with double checkpoint blockade. Based on their response, determined by the increase of the intratumoral CD8+ cells, patients were selected for subsequent radioimmunotherapy [10]. The dataset used in this work consists of tissue sections resulting from the pre-therapeutic biopsies of 101 patients. One slide per patient was used. The slides were stained using hematoxylin and eosin (HE) and digitized as WSIs. Out of 57 patients with oropharyngeal cancer, 30 were HPV-related as determined by p16 expression. Patient characteristics are shown in Fig. 1.

### Data annotation

All 101 WSIs were manually annotated with supervision by an experienced pathologist. Using QuPath [11], an open-source software for whole-slide analysis, tissue regions were annotated in two distinct classes, namely tumor and non-tumor. The tumor class includes both tumor cells and surrounding tumor stroma. Tissues such as normal squamous epithelium, connective tissue, glands, muscle, and fat tissue were annotated as non-tumor. White background, damaged tissue, and large regions of blood or necrosis were not annotated. Furthermore, artifacts such as tissue folds were excluded from the annotation.

### Data preprocessing

**Fig. 2:**
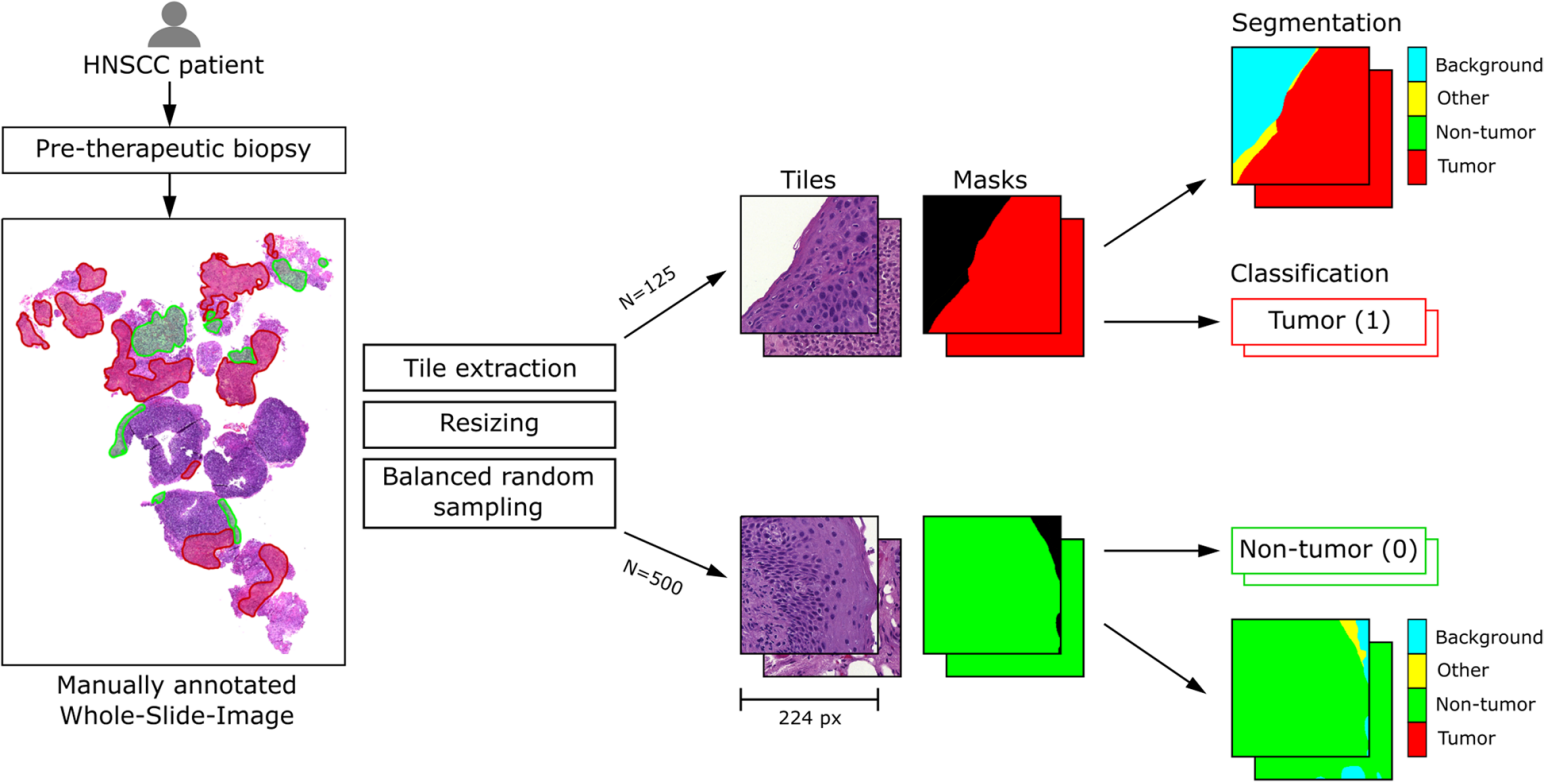
Summary of our data pre-processing pipeline. Each pre-therapeutic WSI was manually annotated. Next, we extracted square tiles from the anntoated tumor (red) and non-tumor (green) regions. Based on the annotations, the ground truth masks for semantic segmentation and the class labels for classification were created

WSIs have very high resolutions and are commonly divided into small square tiles that can be fed to a CNN [12, 13]. The Python library PathML [14] was used to extract non-overlapping tiles with at least 30% annotated pixels. In most slides, more tumor than non-tumor tissue was present and some slides contained no non-tumor tissue. This resulted in a highly imbalanced class distribution. We decided to extract a maximum of 125 tumor and 500 non-tumor tiles from each slide, as depicted in Fig. 2. In this way, the majority class was undersampled and an overall balanced number of tiles per class was achieved  [15, p. 221].

Along with each tile, the corresponding annotations were extracted as binary masks. These masks were further preprocessed for semantic segmentation, as shown in Fig. 2. Specifically, we added a background class for white background pixels and a fourth class for not annotated tissue, in the following referred to as class “other”. The class “other” was added because some tiles contained tissue without any annotation, which should be separated from the background class. The resulting ground truth is a stack of four binary masks, where every pixel belongs to exactly one class. For the classification task, the class with the maximum pixel count was assigned to each tile.

Two important hyperparameters are the tile size, which is commonly between 10 and 250 µm in histopathology [14], and the input resolution, which affects the training speed and accuracy of CNNs. Tile size and resolution influence each other. For example, the impact of varying resolution on the accuracy is greater if images contain more complex information [16]. Thus, both were determined using a grid search with iterated 5-fold cross-validation [17, p. 136] and chosen based on the average validation accuracy of the classification network. In the grid search, the sizes 99.6, 149.4, and 199.1 µm and resolutions ranging from 64 to 512 pixels were considered.

Data augmentation has been shown to improve the generalization of CNNs trained on HE-stained histological images with stain variability [18]. Therefore, several transformations were randomly applied during training, including rotation, mirroring, and variations in hue, saturation, brightness, and contrast. In the training of the classification network, blurring and additive gaussian noise were also applied. The Python library Albumentations [19] was used for transforming tiles and masks jointly. The pixel intensity was scaled to a range of 0 to 1 and then standardized by subtracting the mean value and dividing by the standard deviation of the training data [15, p. 126].

### Deep neural networks

The classification network is an EfficientNet-B0 [20] pre-trained on ImageNet  [21]. We modified this architecture by adding a global average pooling layer, a dense layer with 1024 neurons, Dropout [22], and another dense layer on top. The final dense layer contains a single neuron using a sigmoid activation function. The segmentation network is based on a U-Net architecture [23]. We modified the architecture by replacing the default encoder with EfficientNet-B0 pre-trained on ImageNet [21]. Specifically, the encoder is composed of an input layer and seven blocks of EfficientNet-B0. The decoder consists of five decoder blocks and the output layer, a 1x1 convolutional layer using a softmax activation. A decoder block applies upsampling, followed by two convolutions. Each decoder block is connected to an encoder block by a skip connection. The segmentation network is based on the U-Net architecture with EfficientNet-B0 backbone of the Segmentation Models library [24]. A similar architecture called Eff-Unet with EfficientNet-B7 encoder has been shown to outperform similar approaches [25].

Both networks were implemented and trained in Python using TensorFlow (version 2.8 with Keras API) [26]. The classification network was trained using Adam optimizer [27] to minimize the binary cross-entropy loss. The learning rate was set to $10^{-5}$ and the batch size to 128. The segmentation network was trained using Adam optimizer [27] with a learning rate of $10^{-4}$. We chose to minimize the Jaccard loss function and set the batch size to 64. Additionally, we created two ensemble models. To this end, we converted the predictions of the segmentation network to tile-level predictions. This was implemented by treating the fraction of predicted tumor pixels as tumor probability. The first ensemble model simply averaged the predictions of both networks, which is also called voting. The second ensemble model was a logistic regression model, which was trained on the predictions for the test data using iterated 2-fold cross-validation.

### Explainable AI

For establishing visual interpretability, we relied on two Explainable AI methods that are based on Class Activation Maps (CAMs) [5]. Both methods produce heatmaps, where patterns contributing most to a prediction are highlighted.

Gradient-weighted Class Activation Mapping (Grad-CAM) involves computing the gradient of the class score with respect to feature maps of the final convolutional layer [7]. These feature maps are weighted according to their importance for the predicted class score to produce a coarse localization map [7]. Grad-CAMs can also be created for segmentation networks by replacing the class score by a set of pixels in the output [28]. As recommended by Vinogradova et al., we obtained feature maps from the bottleneck layer [28].

The second method is High-Resolution Class Activation Mapping (HR-CAM) which aggregates feature maps from multiple layers to create a high-resolution localization map [8]. To compute HR-CAMs, the classifier of a frozen CNN is removed, and feature maps are obtained from several convolutional layers. These feature maps are fed to global average pooling and a top dense layer, which is trained to minimize a cross-entropy loss [8]. The heatmap is a weighted sum of the feature maps and the weights of the final dense layer. We obtained feature maps from several layers, as summarized in Table 1. The HR-CAMs for both the classification and segmentation network were created in the same way. We re-trained both models to classify tiles containing at least 80% tumor or 80% non-tumor for 50 epochs.

**Table 1:** Names and output sizes of layers, from which feature maps were obtained. HR-CAM uses feature maps from several layers, whereas Grad-CAM only requires the last convolutional layer’s output. The layer names correspond to the original layer names of EfficientNet-B0 [20]

Method	Classification CNN layers	Segmentation CNN layers	Output size [px]
HR-CAM	block_3a_expand_activation	block_3a_expand_activation	56$\times$56
	block_4a_expand_activation	block_4a_expand_activation	28$\times$28
	block_6a_expand_activation	block_6a_expand_activation	14$\times$14
	block_7a_expand_activation	block_6d_expand_activation	7$\times$7
Grad-CAM	top_activation	block_7a_expand_activation	7$\times$7

## Results

### Dataset compilation

First, we determined the ideal settings for training CNNs. To this extent, square tiles were extracted from the WSIs at 51$\times$ magnification with a pixel size of 194 nm. Using our grid search approach (see Methods), the highest score was reached using a tile size of 199.1 µm (corresponding to 1024 original pixels) and resampled resolutions of 224 to 512 pixels. Therefore, we extracted tiles of size 199.1 µm and resized them to 224 pixels. The scores for different combinations of sizes and resolutions are shown in Additional file 1, Fig. S1. The 101 slides were split into three subsets. 70 slides were used for training, 10 for validation, and 21 for testing. In total, the dataset contained 20,195 tiles.

### CNNs are suited to classify and segment head and neck cancer tissue

**Fig. 3:**
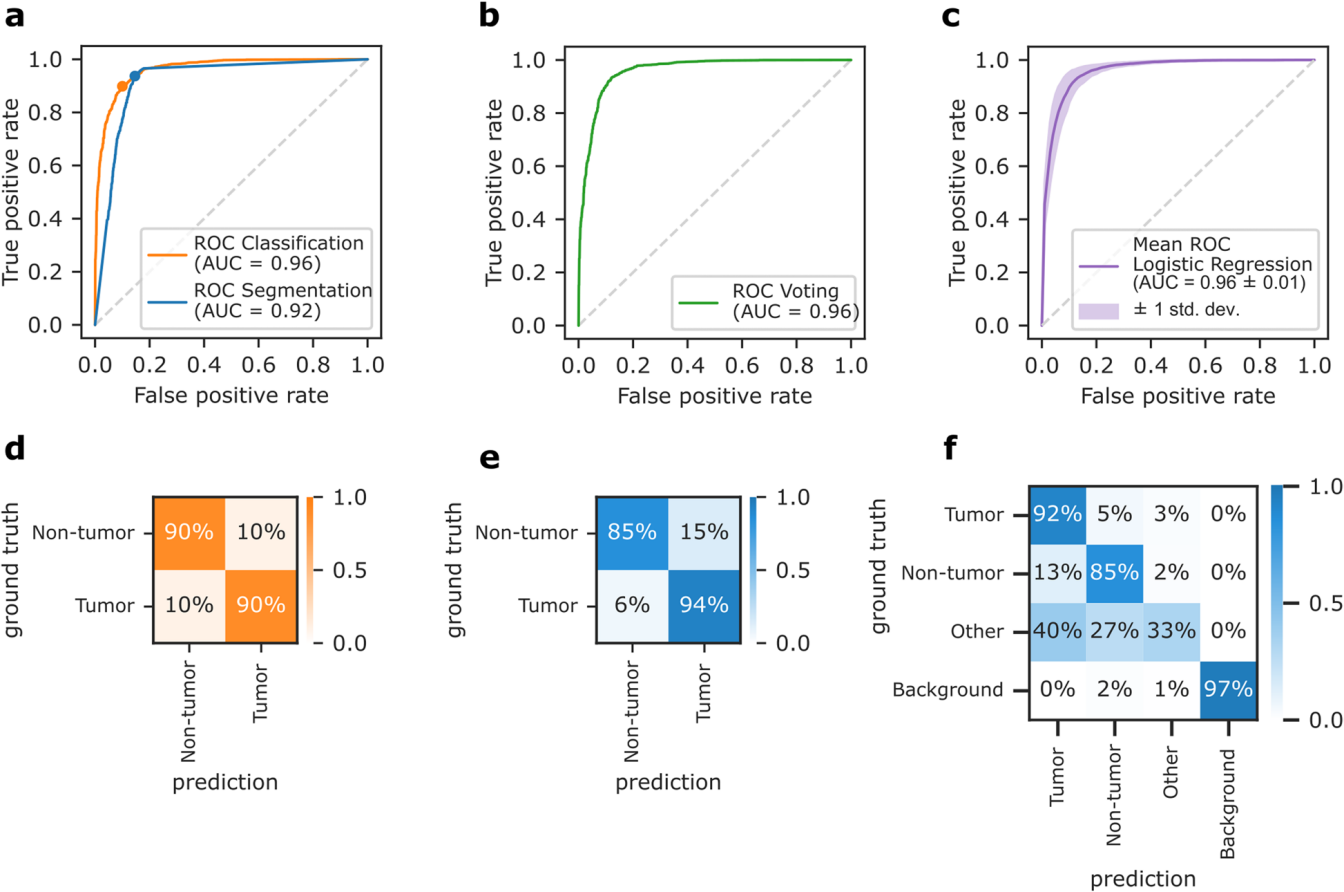
Performance of the classification and segmentation network on test data. **a**-**c** Receiver operating characteristic (ROC) curves and area under the curve (AUC). **a** Comparison of both networks, where the segmentation network’s pixel-level predictions were converted to tile-level predictions. **b** Model ensemble using voting. **c** Model ensemble using logistic regression. **d**-**f** Row-normalized confusion matrices. **d** Classification network. **e** Segmentation network with tile-level predictions. **f** Segmentation network with pixel-level predictions

The networks were evaluated using a previously unseen test dataset of 21 patients. Figure 3 shows their performance in terms of receiver operating characteristic (ROC) curves and confusion matrices. For a better comparison of the two networks, the predictions of the segmentation network were converted to tile-level predictions (see Methods). The resulting ROC curves are shown in Fig. 3a, and the corresponding confusion matrices in Fig. 3d and e. We found that the segmentation network had higher sensitivity but was outperformed by the classification network regarding accuracy and area under the curve (AUC), as summarized in Table 2. The classification network achieved 89.9% and the segmentation network 85.9% accuracy on the test data.

The confusion matrix in Fig. 3f contains the original pixel-wise predictions of the segmentation network. It achieved a class-averaged Jaccard coefficient of 0.690, and 0.782 for the tumor class in particular. Figure 3f shows that only 33% pixels of class “other” were correctly classified. This class contains pixels that were not manually annotated. For example, it includes tissue edges due to imprecise annotations, or artifacts. However, it may also include some tumor or non-tumor tissue, which led to low scores. We next asked if combining the results of the classification and the semantic segmentation CNNs improves the prediction accuracy. We found that neither of the two tested ensemble models, i.e. averaging the predictions or fitting a logistic regression decision function, outperformed the pure classification network, as shown in Fig. 3b-c.

**Table 2:** Quantitative performance evaluation of both networks and two model ensembles. The segmentation networks’ predictions were converted to tile-level predictions in advance for better comparison. All performance metrics were computed for the full test dataset, except for ensemble logistic regression, where the mean values were obtained using iterated 2-fold cross-validation

	Accuracy	AUC	Sensitivity	Specificity
Classification network	89.9%	0.963	89.8%	90.0%
Segmentation network	85.9%	0.921	93.6%	85.4%
Ensemble averaging	87.1%	0.959	86.4%	87.8%
Ensemble logistic regression	89.7%	0.960	91.1%	90.0%

**Fig. 4:**
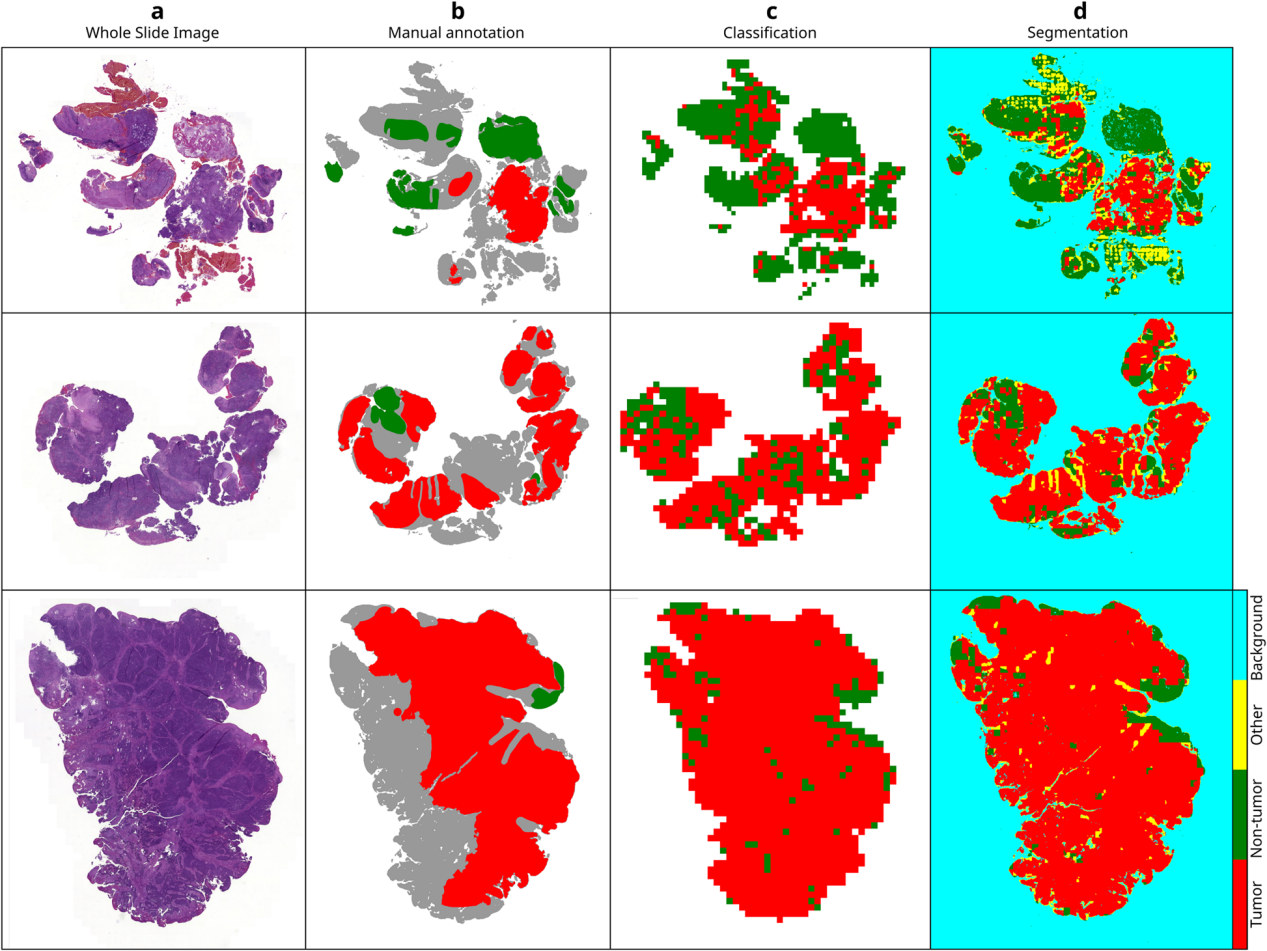
Qualitative assessment of predictions for three WSIs of the test dataset. **a** WSIs. **b** Manual annotation. Not annotated tissue is shown in gray. **c** Predictions of the classification network, created by assigning class labels with a threshold of 0.5 and removing background tiles for better visibility. **d** Predictions of the segmentation network

Both networks yielded predictions for individual tiles. These need to be merged to visualize them for full WSIs. Our workflow for inference and visualization is illustrated in Additional file 1, Fig. S2. To reduce inference time, foreground detection was applied prior to tile extraction. Additionally, we imported predictions into QuPath to enable viewing the tissues and predictions jointly. Figure 4a shows three WSIs of the test dataset, followed by the manual annotation in Fig. 4b and corresponding predictions. To produce the colormaps in Fig. 4c, foreground detection was performed, and the resulting tiles were fed to the classification network. Additionally, the tumor probabilities were converted to binary class labels. The tumor probability map for all tiles, including background tiles, can be found in Additional file 1, Fig. S3. Figure 4d depicts the predictions of the segmentation network. In the segmentation maps, the class with the maximum probability was assigned to each pixel.

### Class Activation Maps highlight pathological patterns

**Fig. 5:**
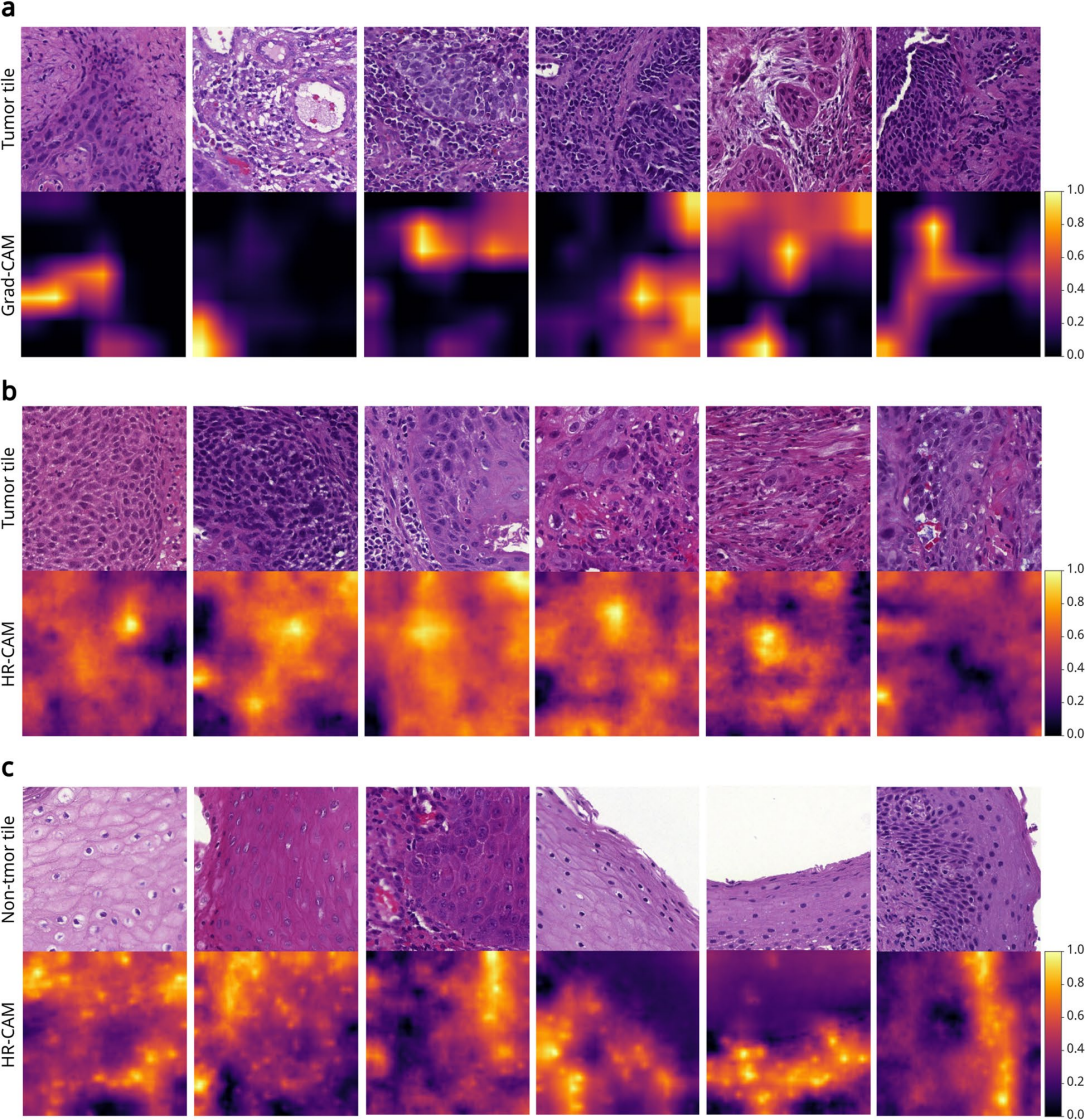
Exemplary tiles of the test dataset with corresponding Grad-CAMs and HR-CAMs, generated for the classification network. The Grad-CAMs (7$\times$7 pixels) were resized to 224$\times$224 pixels. Values close to one indicate a high importance of the respective image region. **a** Tiles containing both tumor and surrounding tissue with Grad-CAMs. **b** Tiles containing atypical cells with HR-CAMs. **c** Tiles containing squamous epithelium with HR-CAMs

Grad-CAMs and HR-CAMs were computed for all tiles of the test dataset. We viewed samples of correct predictions to explore important patterns. First, we examined tiles that were correctly predicted as tumor by the classification network. The Grad-CAMs confirmed that the classifier focused strongly on present tumor cells instead of other surrounding tissue, as shown in Fig. 5a. HR-CAMs of tumor predictions led to the assumption that the presence of atypical cells was an important feature for the classification network. In some samples, a strong focus was on cells characterized by hyperchromatic nuclei that appear dark in the image. Other relevant features might be the abnormal size or irregular shape of the nucleus. These characteristics are also the key features in the decision-making process for pathologists. Examples, where such atypical cells are highlighted, are shown in Fig. 5b. We found that mitotic figures did not seem to be a relevant feature for tumor prediction, although it is a characteristic that pathologists often consider.

Additionally, we looked at the correct predictions of non-tumor samples. The Grad-CAMs appeared to be rather difficult to interpret because it was not clear why specific regions were highlighted. However, the HR-CAMs revealed that neighboring epithelial nuclei, often forming a structured pattern, are an important feature for the classification network. This is shown in Fig. 5c.

We found that Grad-CAMs and HR-CAMs highlighted similar patterns, with a mode correlation of 70.00% for the classification network. The mode correlation of 36.67% for the segmentation network was much lower. The corresponding distributions of the Pearson correlation coefficient can be found in Additional file 1, Fig. S4. To investigate whether decisions of the classification and the segmentation network were based on similar features, the correlation of their CAMs was also computed. We found that CAMs generated for the two distinct networks had a low correlation. In many cases, they focused on different image regions. The mode correlation of Grad-CAMs was 23.33%. HR-CAMs showed a higher mode correlation of 56.67%.

**Fig. 6:**
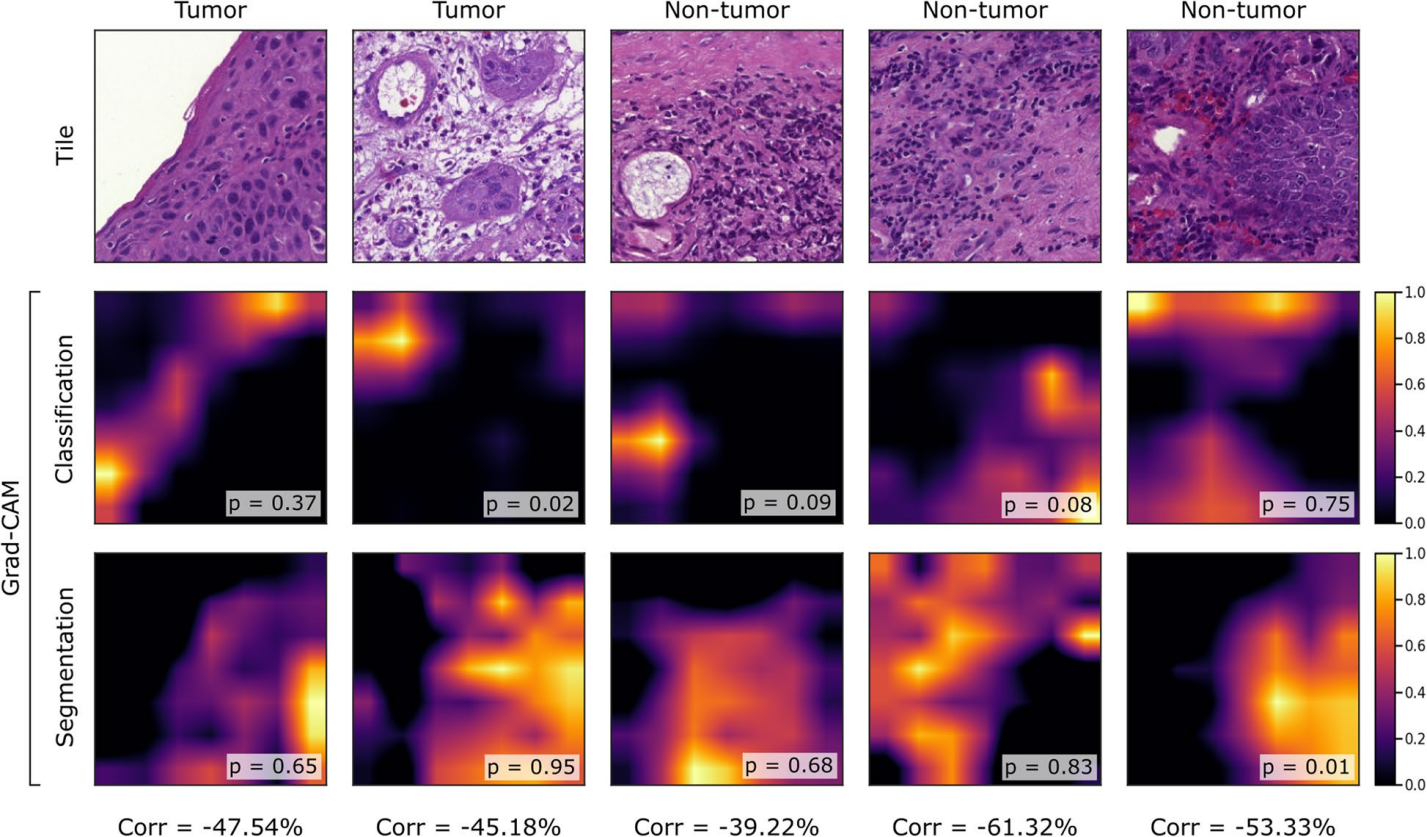
Exemplary test tiles with a negative correlation between Grad-CAMs generated for the classification network and segmentation network. For each pair of Grad-CAMs, the Pearson correlation coefficient (Corr) was computed. For classification, the predicted tumor probability (p) and for segmentation, the fraction of predicted tumor pixels (p) is given. On the top, the binary ground truth labels, i.e. the predominant class in the tile based on the maximum pixel count, are shown

Figure 6 demonstrates the decision-making process as highlighted by Grad-CAM, and why we found different class predictions across the networks. For example, the tile in the first column shows carcinoma. The segmentation network predicted 65% tumor pixels based on present tumor cells. In contrast, the classification network predicted non-tumor because it focused on a region of cells that closely resemble healthy epithelial cells. The example in the second column in Fig. 6 was classified as non-tumor because of the presence of a blood vessel, but the segmentation network detected cancer cells in the tile.

## Discussion

In this work, we created a manually annotated dataset from HE-stained slides of locally advanced HNSCC and trained CNNs for classification and semantic segmentation. Both networks achieved a comparable performance as well as a high accuracy on unseen data, demonstrating their suitability for the detection of head and neck cancer.

The techniques Grad-CAM and HR-CAM were used to create visual explanations. The CAMs showed that both networks learned pathological patterns. For example, the presence of atypical cells with features such as irregular shapes or hyperchromatic, enlarged nuclei seemed to be important. Previously, Grad-CAM has shown that nuclear features contributed most to predictions of head and neck cancer [29] or to predictions of molecular subtypes of muscle-invasive bladder cancer [30]. These features agree with features used by expert pathologists, although they consider more characteristics such as the tissue structure or the number of mitoses, and take additional information into account, such as overall tumor morphology, growth patterns, and tumor architecture. Integrating the detection of such features for model training could be considered, for example, to enable cancer subtyping.

The reliability and meaningfulness of explanations created for the classification network were highlighted by a high correlation between Grad-CAMs and HR-CAMs. We found that the classification network and the segmentation network learned partly overlapping, but yet distinct patterns with a low overall correlation. This suggests taking both architectures into account. We found that Explainable AI techniques were very useful for investigating wrong or differing predictions of the two networks. CAMs can not only help in model development but also assist pathologists in reviewing predictions, making it easier for them to understand the CNN’s abilities and to detect errors [6]. Moreover, it has been shown that presenting Grad-CAMs as additional information along with WSIs can improve the classification accuracy of pathologists [30].

One limitation of our work results from coarse annotations. Most WSIs contained large, connected regions of either tumor or non-tumor tissue. Thus, it was very rare that one tile contained both classes. The segmentation network tended to classify most pixels in a tile either as tumor or non-tumor. This resulted in undesirable, tile-shaped class boundaries, as shown in Fig. 4d. Additionally, Halicek et al. recommend favoring a binary classification task in combination with such coarse annotations [29]. Therefore, the classification network is more suitable than the segmentation network to be trained on our dataset.

Tile-shaped class boundaries were also observed in multi-class breast cancer segmentation by Ho et al. who tackled this problem using multiple magnifications and precise annotations [31]. We argue that our segmentation network could improve using a similar strategy. Moreover, we found that binary class labels were ambiguous when both classes were present in a single tile. For example, the tile in the fifth column of Fig. 6 contained both cancer cells and squamous epithelium. This is a disadvantage of the binary classification approach. To avoid too coarse annotations and ambiguous labels, annotating individual cells should be considered, although this is more costly and time-consuming.

A second limitation is that both networks were not explicitly trained to distinguish tissues and artifacts. We observed that artifacts, such as written text or dust on the slide, were usually classified as non-tumor. However, some artifacts occurred as not annotated regions in the tiles, causing them to be labeled as class “other” by the segmentation network. This applied for tissue-fold artifacts and blood, for example. The left-most WSI in Fig. 4a contains blood, which is classified as “other” in Fig. 4d. An example of tissue-fold artifacts can be found in Additional file 1, Fig. S2. Still, we recommend applying a preceding artifact removal. Alternatively, artifacts could be annotated as an additional class for CNN training.

Another limitation relates to the use of HR-CAM for the segmentation network. HR-CAM relies on adding a global average pooling layer and a dense layer on top of the trained network [8]. The resulting model can only be trained for a classification task and not for semantic segmentation. Most likely, the resulting HR-CAMs highlight features important for the classification task and are therefore biased. This influenced the correlation between HR-CAMs and other CAMs, and led to a skewed distribution of correlation values, as shown in Additional file 1, Fig. S2c-d. For explaining the predictions of a segmentation network, Grad-CAM is more suitable than HR-CAM.

## Conclusion

Our CNNs were able to detect head and neck cancer in unseen slides with high accuracy. This work contributed to the understanding of which features are learned from histological images, by comparing Grad-CAMs and HR-CAMs of the CNNs trained for different tasks. The Explainable AI techniques confirmed that the networks predicted the tumor class based on present pathological patterns, possibly focusing on nuclear features of atypical cells. This is consistent with how pathologists analyze tissue. Thus, CNNs seem promising in assisting pathologists in the assessment of cancer sections, especially in combination with visual explanations.

In the future, we will explore an alternative annotation strategy, namely annotating cell nuclei with a larger number of distinct classes. Future studies may also help to identify more nuclear features such as cell size, nucleoli, and cytoplasmic features in addition to the features we highlighted, to help identify cancer subtypes. Additional Explainable AI techniques such as occlusion experiments [32] or DeepLift [33] could be applied to further study class-discriminative features.

### Supplementary Information

**Additional file 1:** **Figure S1.** Mean validation accuracy for combinations of tile size and tile resolution, resulting from a grid search with iterated 5-fold cross-validation. Tiles were extracted at 512 pixels (99.6 µm), 768 pixels (149.4 µm), and 1024 pixels (199.1 µm), and then resized to different resolutions. **Figure S2.** Inference and visualization of predictions for an exemplary WSI in QuPath. (a) Classification map. (b) Segmentation map. (c), (d) and (e) Sections of both maps, superimposed on the image, in QuPath. Tumor is highlighted in red, non-tumor in green and “other” in yellow. Background tiles were omitted. **Figure S3.** Tumor probability map for three WSIs of the test dataset. (a) WSIs. (b) Manual annotation. (c) Tumor probability map, created from predictions of the classification network for all tiles, including background tiles. **Figure S4.** Correlation of CAMs of the two networks and using the two Explainable AI techniques. (a) Strong correlation between Grad-CAMs and HR-CAMs of the classification network. (b) Low correlation between Grad-CAMs of the classification and segmentation network. (c) Moderate correlation between Grad-CAMs and HR-CAMs of the segmentation network. (d) Bimodal correlation between HR-CAMs of the classification and segmentation network.

## Data Availability

The datasets used and/or analyzed during the current study are available from the corresponding author upon reasonable request due to restricted access to the patient data of the CheckRad-CD8 trial.
