# Supplementary material for: Explainable convolutional neural networks for assessing head and neck cancer histopathology
Source: Diagn Pathol. 2023 Nov 3;18:121. doi: 10.1186/s13000-023-01407-8 (PMC10623808; doi:10.1186/s13000-023-01407-8)
Supplement: Supplementary file 1 — Additional file 1: Figure S1. Mean validation accuracy for combinations of tile size and tile resolution, resulting from a grid search with iterated 5-fold cross-validation. Tiles were extracted at 512 pixels (99.6 µm), 768 pixels (149.4 µm), and 1024 pixels (199.1 µm), and then resized to different resolutions. Figure S2. Inference and visualization of predictions for an exemplary WSI in QuPath. (a) Classification map. (b) Segmentation map. (c), (d) and (e) Sections of both maps, superimposed on the image, in QuPath. Tumor is highlighted in red, non-tumor in green and “other” in yellow. Background tiles were omitted. Figure S3. Tumor probability map for three WSIs of the test dataset. (a) WSIs. (b) Manual annotation. (c) Tumor probability map, created from predictions of the classification network for all tiles, including background tiles. Figure S4. Correlation of CAMs of the two networks and using the two Explainable AI techniques. (a) Strong correlation between Grad-CAMs and HR-CAMs of the classification network. (b) Low correlation between Grad-CAMs of the classification and segmentation network. (c) Moderate correlation between Grad-CAMs and HR-CAMs of the segmentation network. (d) Bimodal correlation between HR-CAMs of the classification and segmentation network. [file 13000_2023_1407_MOESM1_ESM.pdf]

Supplementary Figures

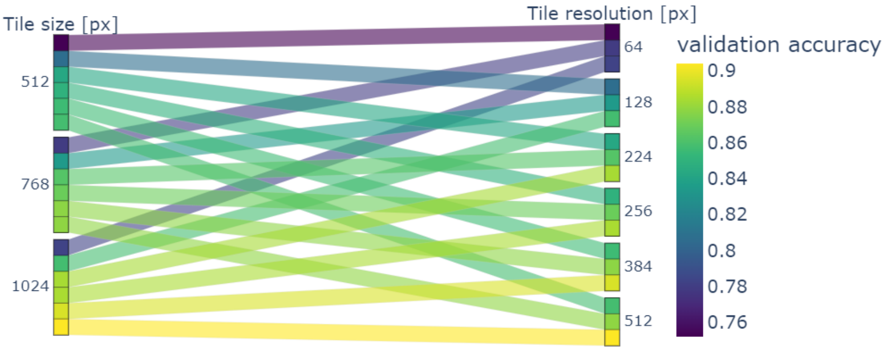

Fig. S1 Mean validation accuracy for combinations of tile size and tile resolution, resulting from a grid search with iterated 5-fold cross-validation. Tiles were extracted at 512 pixels (99.6 µm), 768 pixels (149.4 µm), and 1024 pixels (199.1 µm), and then resized to different resolutions.

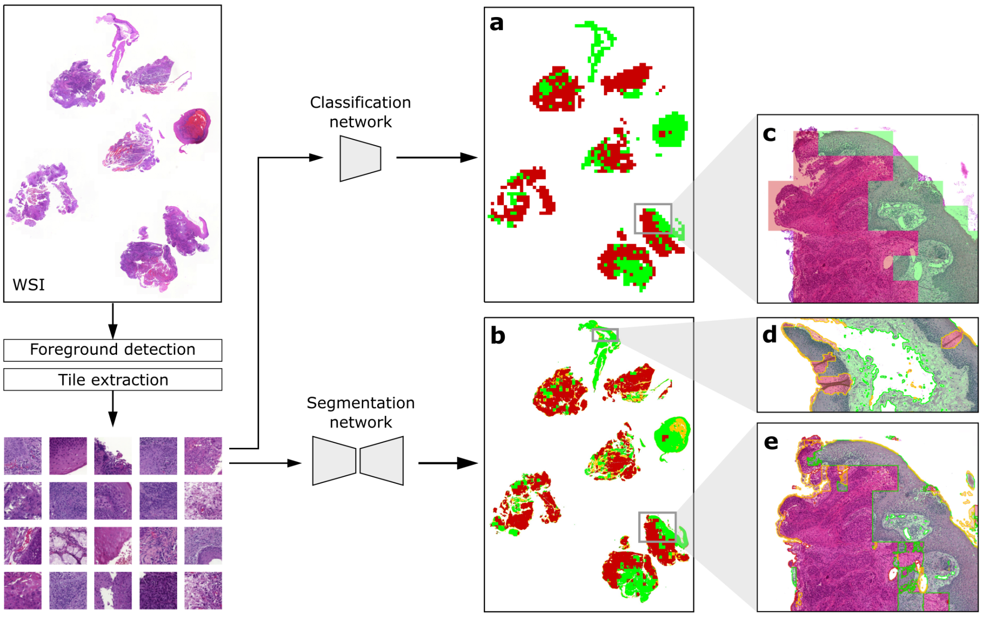

Fig. S2 Inference and visualization of predictions for an exemplary WSI in QuPath. (a) Classification map. (b) Segmentation map. (c), (d) and (e) Sections of both maps, superimposed on the image, in QuPath. Tumor is highlighted in red, non-tumor in green and "other" in yellow. Background tiles were omitted.

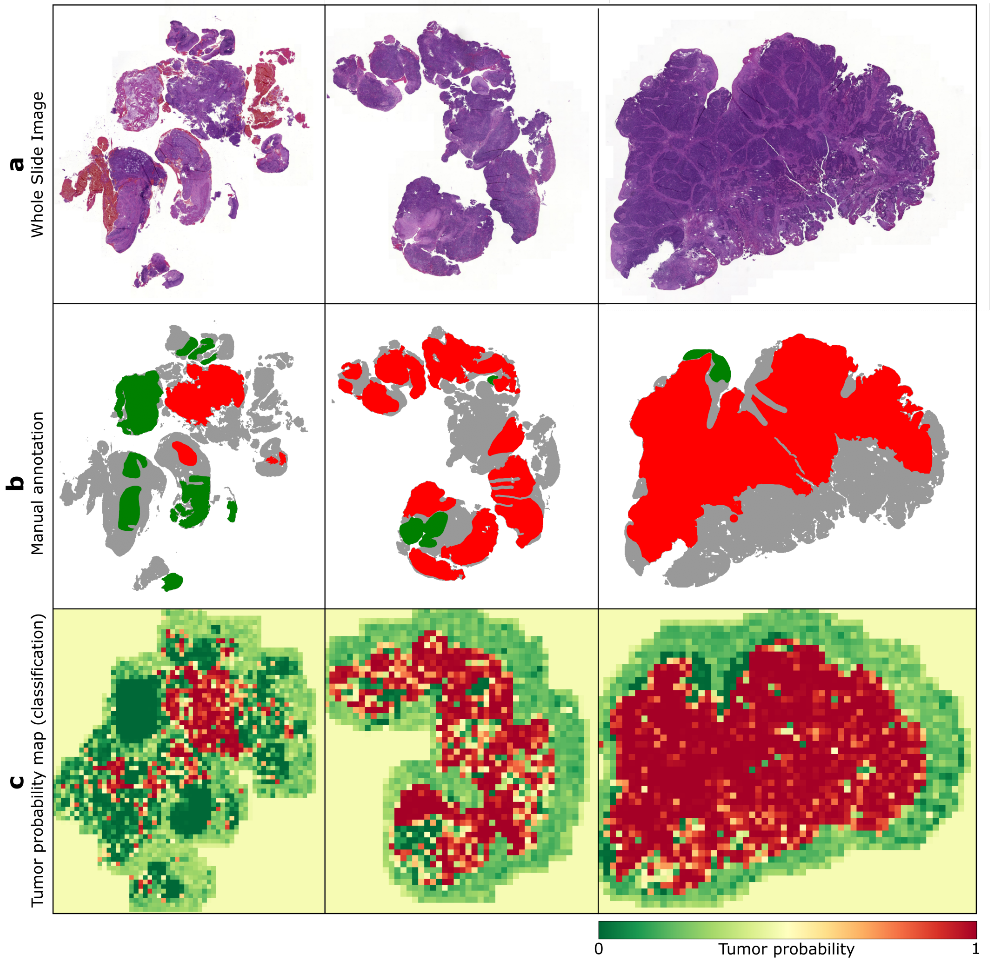

Fig. S3 Tumor probability map for three WSIs of the test dataset. (a) WSIs. (b) Manual annotation. (c) Tumor probability map, created from predictions of the classification network for all tiles, including background tiles.

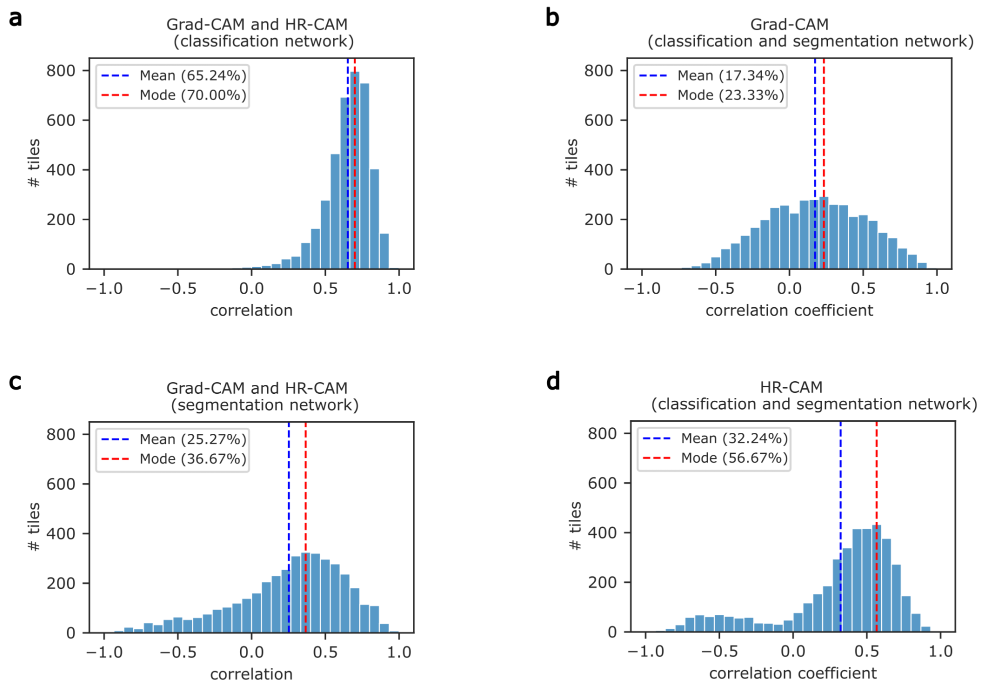

Fig. S4 Correlation of CAMs of the two networks and using the two Explainable AI techniques. (a) Strong correlation between Grad-CAMs and HR-CAMs of the classification network. (b) Low correlation between Grad-CAMs of the classification and segmentation network. (c) Moderate correlation between Grad-CAMs and HR-CAMs of the segmentation network. (d) Bimodal correlation between HR-CAMs of the classification and segmentation network.
